# Epidermal growth factor mediates detachment from and invasion through collagen I and Matrigel in Capan-1 pancreatic cancer cells

**DOI:** 10.1186/1471-230X-5-12

**Published:** 2005-03-31

**Authors:** Andrew J Shirk, Rahul Kuver

**Affiliations:** 1 Division of Gastroenterology, Department of Medicine, University of Washington School of Medicine, and the Puget Sound Veterans Administration Health Care System, Seattle Division, Seattle, Washington USA

## Abstract

**Background:**

Pancreatic adenocarcinoma is a highly invasive neoplasm. Epidermal growth factor (EGF) and its receptor are over expressed in pancreatic cancer, and expression correlates with invasion and metastasis. We hypothesized that EGF receptor and integrin signalling pathways interact in mediating cellular adhesion and invasion in pancreatic cancer, and that invasiveness correlates temporally with detachment from extracellular matrix.

**Methods:**

We tested this hypothesis by investigating the role of EGF in mediating adhesion to and invasion through collagen I and Matrigel in the metastatic pancreatic adenocarcinoma cell line Capan-1. Adhesion and invasion were measured using *in vitro *assays of fluorescently-labeled cells. Adhesion and invasion assays were also performed in the primary pancreatic adenocarcinoma cell line MIA PaCa-2.

**Results:**

EGF inhibited adhesion to collagen I and Matrigel in Capan-1 cells. The loss of adhesion was reversed by AG825, an inhibitor of erbB2 receptor signalling and by wortmannin, a PI3K inhibitor, but not by the protein synthesis inhibitor cycloheximide. EGF stimulated invasion through collagen I and Matrigel at concentrations and time courses similar to those mediating detachment from these extracellular matrix components. Adhesion to collagen I was different in MIA PaCa-2 cells, with no significant change elicited following EGF treatment, whereas treatment with the EGF family member heregulin-alpha elicited a marked increase in adhesion. Invasion through Matrigel in response to EGF, however, was similar to that observed in Capan-1 cells.

**Conclusion:**

An inverse relationship exists between adhesion and invasion capabilities in Capan-1 cells but not in MIA PaCa-2 cells. EGF receptor signalling involving the erbB2 and PI3K pathways plays a role in mediating these events in Capan-1 cells.

## Background

Pancreatic cancer carries a poor prognosis due to the advanced level of tumor invasion and metastasis often encountered at the time of diagnosis [1]. A clear understanding of the cellular processes involved in pancreatic cancer invasiveness and metastasis may be useful in the development of novel forms of therapy. The receptor tyrosine kinase epidermal growth factor receptor (EGF-R) and its ligands are over expressed in pancreatic cancer tissues and in pancreatic cancer cell lines [2,3], with coexpression of receptor and ligand correlating with tumor invasiveness [4]. The mitogenic effects of EGF-R stimulation in pancreatic cancer are well established [5,6]. The importance of EGF-R signalling in promoting and maintaining pancreatic cancer growth is highlighted by studies showing decreased growth in mice treated with a receptor tyrosine kinase blocker [7] and by an EGF-R blocking antibody [8,9].

In contrast to the role of EGF-R and its ligands on cell proliferation, the mechanisms involved in EGF-R mediated invasiveness in pancreatic cancer cells are unclear. Specifically, the role of EGF-R signalling with respect to cellular adhesion to extracellular matrix, cellular motility and invasion through extracellular matrix in pancreatic cancer cells are not known. The involvement of EGF-R and related erbB receptor tyrosine kinases in cancer cell invasion is suggested by studies in colon and mammary carcinoma cell lines. In colon cancer cells, EGF-R activation correlates with the development of liver metastases [10,11]. In breast cancer cells, heregulin-alpha (HRG-α), an EGF family member that binds to erbB3 and erbB4 and facilitates heterodimerization of these receptors with the erbB2 receptor, regulates the actin cytoskeleton and promotes invasion [12]. Signalling via the erbB family of receptor tyrosine kinases therefore is likely to play an important role in mediating pancreatic cancer invasiveness as well.

A combination of signals from cytokine, growth factor and adhesion receptors regulates cell motility. In cancer cells, motility may become dysregulated leading to increased invasive potential. A model of cancer cell invasion would include the loosening of attachment to the primary tumor mass in a process involving detachment from extracellular matrix. The detached cells would then penetrate the extracellular matrix thereby leading to cell migration and invasion. Because EGF and related erbB receptor ligands have been implicated in cellular adhesion and migration [13-17], we set out to investigate the role of EGF in adhesion and invasion in a pancreatic cancer cell line. We chose Capan-1 cells, as these are well-differentiated cells originally isolated from liver metastases in a patient with pancreatic cancer [18]. We also performed adhesion and invasion studies on MIA PaCa-2 cells, which were originally isolated from a primary pancreatic adenocarcinoma, to determine whether adhesion and invasion characteristics were shared between these cells and Capan-1 cells.

## Methods

### Materials

Eagle's minimum essential media (EMEM), FBS, trypsin-EDTA, penicillin-streptomycin, phalloidin-TRITC, fibronectin, collagen I, laminin IV, AG825, wortmannin and Cell Dissociation Solution were from Sigma (St. Louis, MO). RPMI 1640 media was from GIBCO BRL (Grand Island, NY). Matrigel was from Becton Dickenson Biosciences (Bedford, MA). Recombinant human EGF was from Calbiochem (San Diego, CA). Recombinant heregulin-α was from R&D Systems (Minneapolis, MN). Vitrogen was from Celtrix (Palo Alto, CA). Transwell cell culture plates (diameter 24 mm; pore size 3.0 μm) were from Costar (Cambridge, MA). Six well and 96 well plates were from Becton Dickinson (Franklin Lakes, NJ). Enhanced chemiluminescence detection reagents (ECL-Plus) were from Amersham Pharmacia (Piscataway, NJ). Calcein-AM, To-Pro-3 iodide and fluorescein-conjugated goat anti-rabbit IgG were from Molecular Probes (Eugene, OR). The anti-human mouse monoclonal EGF-R and the anti-human rabbit polyclonal EGF antibodies was from Oncogene (Boston, MA). Anti-human mouse monoclonal erbB2 and erbB3 antibodies were from NeoMarkers (Fremont, CA). For western blotting, α2 integrin and β1 integrin anti-human mouse monoclonal antibodies were from Transduction Laboratories (Lexington, KY). The rabbit anti-human GAPDH antibody was from Novus (Littleton, CO). The human EGF ELISA kit was from Biomedical Technologies (Stoughton, MA).

### Cell lines and cell culture

Capan-1 cells were obtained from American Type Culture Collection (Manassas, VA) and cultured in RPMI 1640 supplemented with 15% FBS and 2 mM L-glutamine. MIA PaCa-2 cells were also obtained from American Type Culture Collection and cultured in Dulbecco's modified Eagle medium supplemented with 10% FBS and 4 mM L-glutamine. Cells were passaged when confluent. Once confluent, cells were switched over to serum free media gradually over three successive feedings on alternate days, then treated with growth factors in serum free media for the indicated time periods. Human fibroblasts were derived from human gallbladders and cultured on six-well plates as previously described [19].

### Adhesion assay

Adhesion assays were conducted in 96 well plates coated overnight at 4°C with 10 μg/ml collagen I, Matrigel, laminin IV, fibronectin, hyaluronic acid or 1% BSA. After coating, the wells were incubated with 1% BSA in PBS for 1 hour at 37°C, then washed with PBS before use. Cells were labelled for 1 hour with 1 mM Calcein AM at 37°C, washed with PBS, and then detached with 5 mM EDTA in PBS or with Cell Dissociation Solution. Detached cells were washed once more in PBS, resuspended in serum free media and added to the wells (5 × 10^4 ^cells per well). The plates were then incubated at 37°C for 1 hour. Each well was then washed three times in PBS, and then overlaid with serum free media prior to quantification. The percentage of adherent cells was determined using a Cytofluor 4000 fluorescence plate reader (Applied Biosystems, Foster City, CA), with excitation wavelength 485 nm and emission wavelength 530 nm. Results are expressed as the ratio of fluorescence from wells containing matrix to uncoated control wells containing 5 × 10^4 ^cells.

For the adhesion assays using inhibitors, growth factor treatment duration was 8 hours. Cells were also treated with the indicated inhibitors at the following final concentrations and durations of preincubation prior to addition of growth factor: cycloheximide 10 μM for 30 minutes; wortmannin 100 nM in DMSO for 60 minutes; AG825 25 μM in DMSO for 60 minutes; erbB2 and erbB3 blocking antibodies 10 μg/ml for 30 minutes. Initial experiments were performed with a range of doses, with the indicated doses being the ones for which data are shown. Inhibitors were present throughout the duration of exposure to growth factor or vehicle. Cell toxicity was monitored using trypan blue exclusion during incubations with inhibitors, with no treatment leading to >5% cell death.

### Invasion assay

Invasion assays were performed using a modification of a previously described method [20]. Briefly, 8 μM pore HTS Fluoroblok filters (Becton Dickenson) were coated overnight with 300 μl Matrigel or collagen I diluted to 100 μg/ml in PBS at 4°C. The matrix was rehydrated for two hours with serum free media prior to use, then washed once with PBS. Cells were labelled for 1 hour with 1 mM Calcein AM at 37°C, washed with PBS, and then detached with 5 mM EDTA in PBS or with Cell Dissociation Solution. Detached cells were washed once more in PBS, resuspended in serum free media and added to the invasion chamber (1 × 10^5 ^cells per well). The chambers were incubated at 37°C for up to 48 hours. The percent of cells invading to the bottom of the membrane was then quantified using a Cytofluor 4000 fluorescence plate reader. Results are expressed as the ratio of fluorescence from cells invading to control wells containing 1 × 10^5 ^cells.

### Confocal laser scanning immunofluorescence microscopy

Cells were cultured in 24 mm diameter Transwell inserts until confluent. Cells were washed twice with ice cold PBS, then a square of membrane was excised. This square was then fixed in methanol/acetone (1:1; v/v) for 15 minutes at -20°C. The fixed cells were then washed in ice cold PBS and incubated for 30 minutes in the presence of PBS containing 1% BSA. The cells were then washed in PBS and incubated in primary antibody diluted in PBS containing 1% BSA. After one hour, the cells were again washed and incubated with secondary antibody in PBS containing 1% BSA. The secondary antibody was removed after one hour by washing with PBS. To-Pro-3 iodide was applied for 1 hour. Cells were washed three times with PBS and sealed in Vectashield Hard Set mounting medium (Vector Labs, Burlingame, CA). Scanned images were acquired with 400× optical and 3× digital magnification using a laser scanning spectral confocal microscope system (Leica DM-R upright fluorescence microscope and Leica TCS-SP confocal scanner). Single serial section images in the xy plane were acquired. Cells treated with secondary antibody only did not show detectable signals at the parameters used for acquiring images.

### Immunoblotting

Cells were cultured to confluency on six well plates and harvested with SDS loading buffer (250 mM Tris, ph 6.8; 4% SDS; 10% glycerol; 0.006% bromphenol blue; and 2% β-mercaptoethanol). Protein content of cell extracts was measured by the Lowry method. SDS-PAGE was performed followed by transfer to PVDF membrane. The membranes were blocked with 10% BSA in PBS/Tween 20 (0.05%; v/v) at 4°C for 16 hours and then incubated with the anti-α_2 _integrin or β_1 _integrin antibodies for 1 hour at room temperature. The membrane was then washed with 0.05% Tween 20 in PBS and incubated with peroxidase-conjugated anti-mouse IgG antibody for 1 hour at room temperature. The membrane was washed and incubated with ECL-Plus and autoradiography performed. We also ran blots in parallel that were hybridized with rabbit anti-human GAPDH antibody to confirm equal protein loading.

### ELISA assay

Capan-1 cells and human gallbladder myofibroblasts were cultured until confluent in 6 well plates. Upon achieving confluency, cells were then cultured in media containing 10% FBS or in serum free media. Following 48 hours of incubation, media was collected, centrifuged to pellet cell debris and floating cells, and soluble EGF levels quantitated using a human EGF ELISA kit, using 96 well plates and a microplate fluorescence reader per the manufacturer's instructions. The sensitivity of this method was to 0.5 ng/ml soluble EGF.

### Statistical analysis

A minimum of three separate experiments (with each treatment condition performed in triplicate) was performed for the adhesion and invasion assays. Results of experiments are expressed as the mean percentage ± SD of cells that are adherent or invading compared to cells from control wells. Student's t test was used, and p < 0.05 was considered significant.

## Results

### Capan-1 adhesion to collagen I is inhibited by EGF

**Figure 1:**
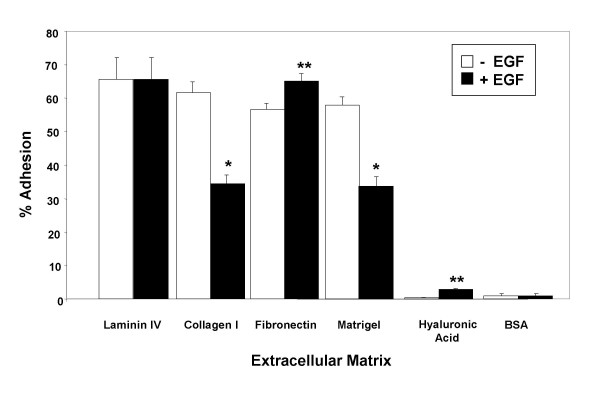
**Adhesion of cells to various extracellular matrix components and the effect of EGF. **Adhesion was tested under conditions of no growth factor (open symbols) and treatment with 10 nM EGF (closed symbols) for 8 hours. Results are expressed as % adhesion ± SEM from three separate experiments, each performed in triplicate wells. * p < 0.003; ** p < 0.05.

We first examined the ability of Capan-1 cells to adhere to various extracellular matrix components. As shown in Figure 1, Capan-1 cells adhered to a variety of matrices, including laminin IV, fibronectin, collagen I and Matrigel. Capan-1 cells did not adhere to any significant degree to hyaluronic acid or to BSA.

We then determined the effects of EGF on adhesion to these extracellular matrix components. For this set of experiments, EGF treatment was provided for 8 hours prior to the initiation of the adhesion assay. Adhesion to collagen I decreased upon treatment with EGF. Similarly, adhesion to Matrigel decreased with EGF treatment. In contrast, treatment with EGF increased adhesion to fibronectin. There was no difference in adhesion to laminin IV. Finally, even though adhesion to hyaluronic acid was low at baseline, EGF treatment increased adhesion. No significant change in adhesion was noted on BSA with EGF treatment. EGF therefore has varied effects on adhesion to various extracellular matrix components, mediating detachment from collagen I and Matrigel, while potentiating adhesion to fibronectin and hyaluronic acid.

Heregulin-α (HRG-α) belongs to the same family as EGF, binds to erbB3 and erbB4 receptors and is capable of heterodimerization with the erbB2 receptor in other cell types [12]. We therefore used HRG-α as an indirect means to determine whether the EGF-mediated effects on adhesion were shared by activation of erbB3 and erbB4 receptors. We tested the effects of HRG-α on the adhesion of Capan-1 cells to the same set of extracellular matrix components and for the same duration of treatment. In contrast to the results with EGF, there was no significant inhibition of adhesion to collagen I with HRG-α treatment. In addition, HRG-α did not alter adhesion to laminin IV, fibronectin, hyaluronic acid or BSA. There was a significant decrease in adhesion to Matrigel (from 57.95 % ± 2.5 to 33.6 % ± 2.9; p = 0.003) with HRG-α treatment. These results imply that the inhibition of adhesion to collagen I occurred without involvement of erbB3 or erbB4 receptors. The inhibition of adhesion to Matrigel, however, appeared to involve receptors activated by either EGF or HRG-α.

### Evidence that the EGF-mediated detachment from collagen I in Capan-1 cells involves erbB1 and erbB2 receptors and the phosphatidylinositol-3 kinase signalling pathway

**Figure 2:**
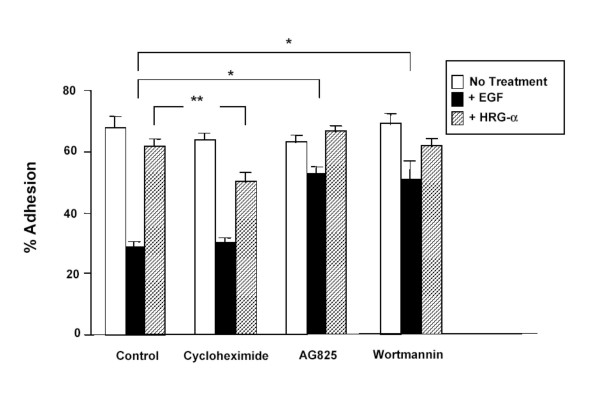
**Effects of various inhibitors on adhesion of cells to collagen I. **Adhesion assays on collagen I were performed with no growth factor (open bars), 10 nM EGF (closed bars) or 10 nM HRG-α (hatched bars). Shown are the results of three separate experiments, each performed in triplicate. * p < 0.001. **p < 0.02.

We focused on adhesion to collagen I by Capan-1 cells given the marked inhibition of adhesion seen with EGF. In order to begin to delineate the pathways involved in this detachment, we examined the effect of various inhibitors: cycloheximide, a protein synthesis inhibitor; wortmannin, an inhibitor of phosphatidylinositol-3 kinase; AG825, a pharmacologic inhibitor of the erbB2 receptor; a blocking antibody against the erbB2 receptor; and a blocking antibody against the erbB3 receptor. Each of these inhibitors did not alter adhesion to collagen I to any significant degree in the absence of growth factor treatment (Figure 2). EGF-mediated de-adhesion to collagen I was not reversed by cycloheximide, suggesting that new protein synthesis was not involved. Similarly, treatment with a blocking antibody against the erbB3 receptor did not alter this EGF-mediated detachment. On the other hand, treatment with wortmannin reversed the EGF-mediated detachment, implying a phosphatidylinositol-3 kinase mediated pathway was involved (EGF = 30.5 % ± 2.1 vs. EGF + wortmannin = 50.5 % ± 3.8; p < 0.001; n = 10). Similarly, an inhibitor of the erbB2 receptor, AG825 and a blocking antibody against erbB2 reversed the EGF-mediated detachment (to 49.9 % ± 1.7; p = <0.001; n = 10; and to 48.6 % ± 2.4; = <0.001; n = 5, respectively). The similar magnitude of this inhibition of the EGF-induced detachment suggested that the erbB2 receptor signalling pathway and the phosphatidylinositol-3 kinase pathway were involved simultaneously.

We also examined the effects of this panel of inhibitors on cells adhering to collagen I and treated with HRG-α. There was no effect on adhesion to collagen I with HRG-α treatment, a finding that was not altered with treatment with wortmannin or the erbB2 receptor inhibitor AG825 (Figure 2). Furthermore, blocking antibodies against the erbB2 and erbB3 receptors did not alter this lack of effect of HRG-α on adhesion to collagen I (data not shown). Cycloheximide decreased adhesion in the presence of HRG-α, (from 57.0 % ± 1.6 to 50.4 % ± 2.1; p = 0.02; n = 10), suggesting that protein synthesis was involved to a limited degree in maintaining adhesion to collagen I with HRG-α treatment. Overall, these results suggested that the detachment from collagen I was an EGF-specific effect, which was not shared by the related growth factor, HRG-α. This de-adhesive effect is therefore likely mediated through activation of the EGF-R and partially through activation of the erbB2 receptor, and involves the phosphatidylinositol-3 kinase signalling pathway.

### Lack of a de-adhesion response to Collagen I of EGF in a primary pancreatic cancer cell line (MIA PaCa-2)

**Figure 3:**
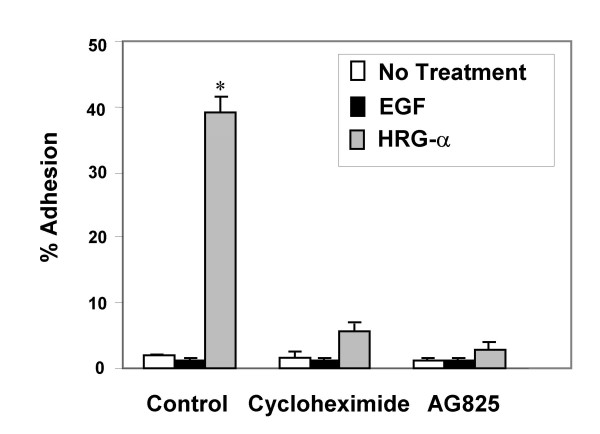
**Effects of cycloheximide and AG825 on adhesion of MIA PaCa-2 cells to collagen I. **Adhesion assays on collagen I using were performed with no growth factor (open bars), 10 nM EGF (closed bars) or 10 nM HRG-α (hatched bars). Shown are the results of three separate experiments, each performed in triplicate. * p < 0.001.

MIA PaCa-2 cells are derived from a primary, non-metastatic pancreatic adenocarcinoma, unlike Capan-1 cells, which were isolated from liver metastases. We therefore asked whether the adhesion response to EGF on collagen I was a property shared between these two pancreatic cancer cell lines. Adhesion to collagen I was markedly diminished in MIA PaCa-2 cells under control conditions compared to Capan-1 cells. Treatment with EGF (10 nM for 8 hours) did not show a significant change in adhesion. Strikingly, however, treatment with HRG-α showed a marked increase in adhesion to collagen I (Figure 3). This effect of HRG-α was abrogated by pre-treatment with either cycloheximide or the erbB-2 inhibitor, AG825, suggesting that protein synthesis and activation of the erbB-2 receptor were involved in the HRG-α response. HRG-α, and by implication, activation of the erbB3 and/or erbB4 receptors in concert with erbB-2 receptor activation, appears to mediate adhesion to collagen I in this cell line.

### EGF stimulates invasion through collagen I and Matrigel in Capan-1 cells

**Figure 4:**
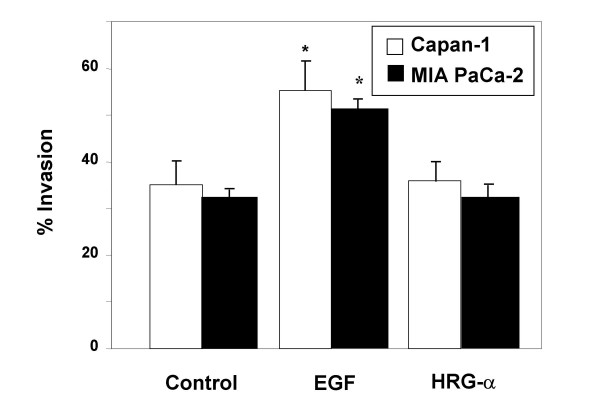
**Effects of EGF and HRG-α on invasion through Matrigel in Capan-1 and MIA PaCa-2 cells. **Cells were cultured on invasion chambers coated with Matrigel, and invasion measured following treatment with no growth factor, 10 nM EGF, or 10 nM HRG-α. Shown are the results of 3 experiments, each performed in triplicate wells. *p < 0.05 between the no growth factor group and the EGF treatment group for each cell type.

We next examined the effects of EGF on invasion through collagen I and Matrigel. We chose collagen I because of the clear detachment response to EGF noted above. We also chose to examine invasion through Matrigel, as this is a more complex matrix that simulates extracellular matrix *in vivo *and serves as a more physiologic substrate on which to measure invasion capabilities of Capan-1 cells. Furthermore, the studies on adhesion had shown parallel effects with EGF treatment, suggesting that collagen I and Matrigel possessed similar properties with respect to adhesion and invasion. Capan-1 cells showed an increase in invasion following addition of EGF which was seen with both collagen I (data not shown) and with Matrigel (Figure 4). No effect on invasion through either collagen I or Matrigel was seen with HRG-α.

### EGF stimulates invasion through Matrigel in MIA PaCa-2 cells

We also assessed invasion through Matrigel with and without growth factor treatment on MIA PaCa-2 cells. As shown in Figure 4, EGF treatment enhanced invasion to a similar extent as in Capan-1 cells, whereas HRG-α had no effect. Therefore, the de-adhesive response to EGF is absent in the MIA PaCa-2 cells, whereas the invasive response to Matrigel is preserved.

### Time course of EGF-mediated inhibition of adhesion and stimulation of invasion

**Figure 5:**
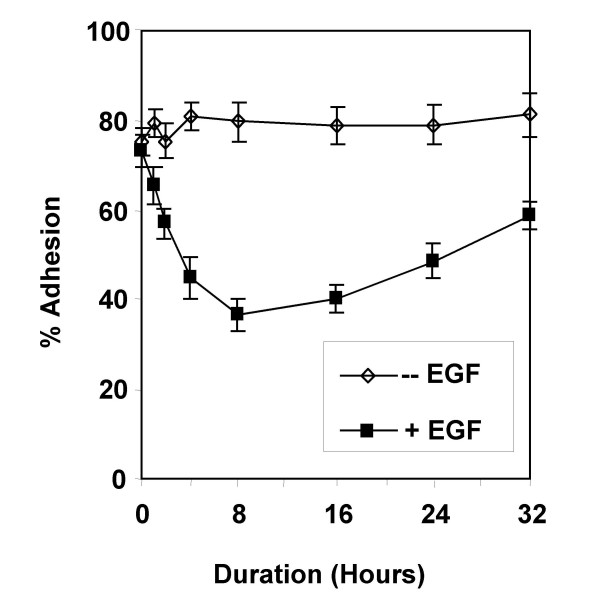
**Time course of EGF-mediated inhibition of adhesion. **The adhesion assay was performed and the experiment terminated at various time points. Adhesion was measured without and with EGF treatment. Shown are the results of one experiment performed in triplicate wells, which was repeated twice with similar results.

Given the EGF-mediated effects on both adhesion and invasion, which were not seen with HRG-α, erbB1/erbB2 receptor signalling pathways were likely involved in mediating both detachment from extracellular matrix and the stimulation of invasion noted in Capan-1 cells. We explored the effects of EGF signal duration on detachment and invasion in Capan-1 cells. EGF treatment led to a time-dependent detachment from collagen I, which was slowly reversible (Figure 5). The peak inhibition of adhesion occurred with 8 hours of EGF treatment.

**Figure 6:**
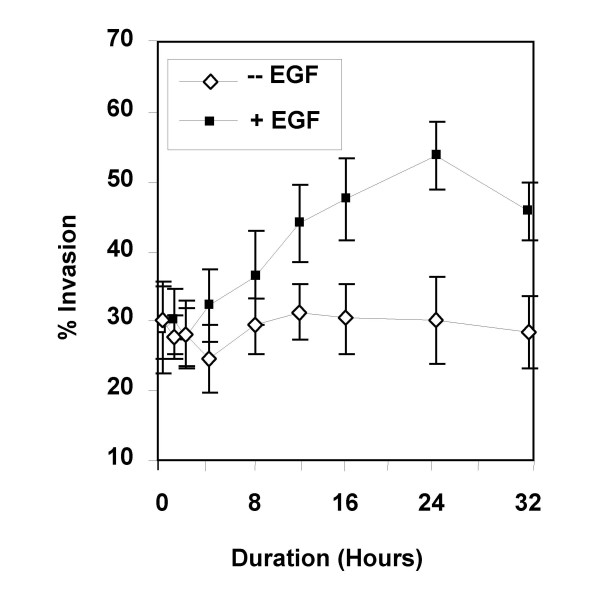
**The time course of EGF-mediated invasion through Matrigel. **Cells were labeled and invasion through chambers performed in the absence or presence of 10 nM EGF. Shown are the results of one experiment performed in triplicate wells, which was repeated twice with similar results.

The time course of invasion through Matrigel showed that the EGF-mediated increase in invasion peaked at 24 hours of treatment, and then began to slowly return to pre-treatment levels (Figure 6). Therefore, the EGF-mediated inhibition of adhesion was followed by an EGF-mediated increase in invasion, suggesting a temporal relationship between loss of attachment to extracellular matrix and acquisition of the ability to invade.

### Dose response of EGF-induced detachment from collagen I and invasion through Matrigel

**Figure 7:**
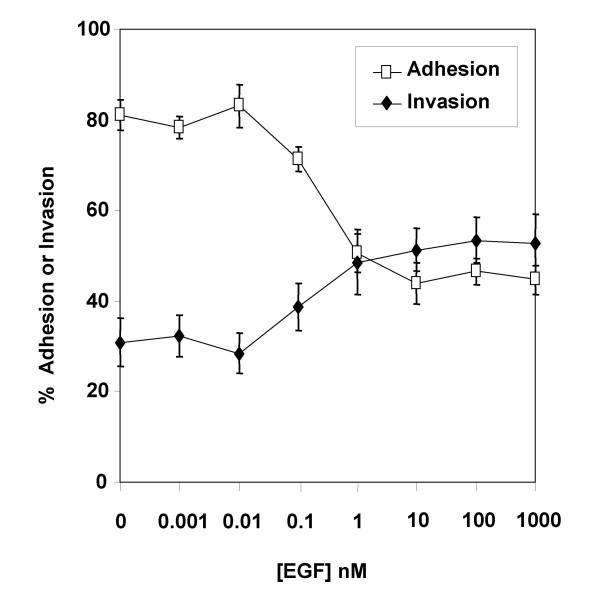
**Dose response of EGF-induced detachment from collagen I and stimulation of invasion through Matrigel. **Adhesion and invasion were measured following incubation with escalating doses of EGF. Shown are the results of three experiments, each performed in triplicate.

This temporal relationship was supported by the finding that the EGF dose response for both adhesion and invasion were similar (Figure 7). Concentrations greater than 0.01 nM were required to see effects on both of these phenomena. For these studies, the duration of treatment with EGF was held constant at 24 hours, a time when both detachment and invasion were statistically significantly different between treated and non-treated cells. The dose of EGF chosen for the earlier assays on adhesion and invasion (10 nM) was justified by these dose response studies, as at that concentration a plateau of both detachment and invasion were seen.

### Expression of EGF receptor, α_2 _integrin and β_1 _integrin in Capan-1 cells

**Figure 8:**
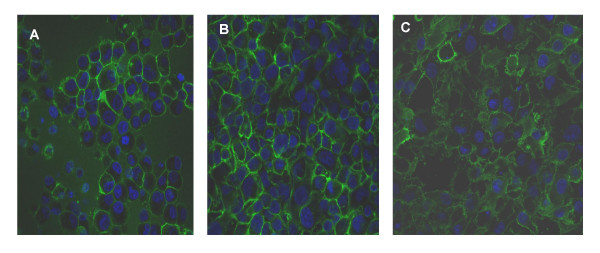
**Expression of EGFR, α_2_/β_1 _integrin in Capan-1 cells. **Cells were cultured on Transwell inserts, and confocal immunofluorescence microscopy performed following incubation with primary antibodies against EGF-R (A), α_2 _integrin (B) and β_1 _integrin (C). Negative controls with primary antibody omitted did not show a detectable signal.

EGF effects are mediated predominantly through the EGF receptor (EGF-R). EGF-R expression was demonstrated in Capan-1 cells by confocal immunofluorescence microscopy (Figure 8A). As effects of adhesion and invasion were likely to involve integrins, we sought to demonstrate expression of the integrins involved in adherence to collagen I, the α_2 _and β_1 _integrins. As shown in Figure 8B, α_2 _integrin was expressed on the cell surface of Capan-1 cells. Similarly, the β_1 _integrin was expressed on the surface of Capan-1 cells (Figure 8C). These results demonstrate that the EGF-mediated effects of adhesion and invasion in Capan-1 cells were likely to involve the EGF receptor, as well as the collagen I receptor, the α_2_β_1 _integrin heterodimer.

We next asked whether α_2_/β_1 _integrin expression levels were affected by EGF treatment. No significant difference in signal for either α_2 _integrin or for β_1 _integrin was noted, either by immunofluorescence or immunoblot analysis, following treatment with 10 nM EGF (data not shown).

### EGF treatment induces rearrangements in the actin cytoskeleton

**Figure 9:**
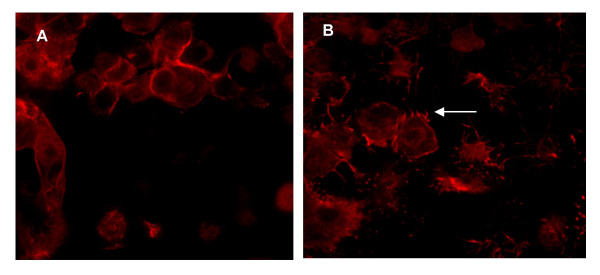
**Phalloidin-TRITC staining of cells with and without EGF treatment. **Cells were cultured on Transwell inserts, and confocal immunofluorescence microscopy performed following incubation with TRITC-conjugated phalloidin, in the absence (A) or presence (B) of 10 nM EGF. Arrow shows focal area of actin staining consistent with a focal adhesion complex.

ErbB1/erbB2 receptor tyrosine kinase signalling mediated by EGF binding has been linked to integrin expression via effects on the focal adhesion complex [21]. One important downstream effect of changes in the focal adhesion complex is actin cytoskeleton rearrangement. Therefore, we looked for changes in actin cytoskeleton architecture using phalloidin staining in cells treated with EGF. As shown in Figure 9, phalloidin staining showed focal areas of staining suggestive of focal adhesion complexes in cells treated with EGF compared to untreated cells.

### Expression of EGF in Capan-1 cells does not correlate with EGF secretion

**Figure 10:**
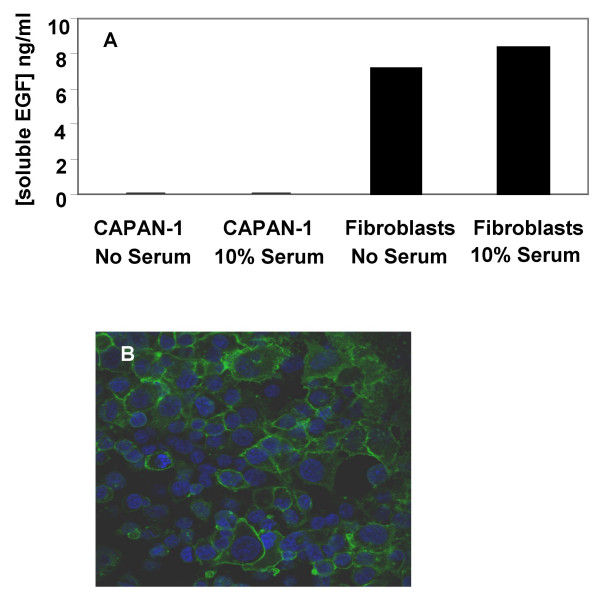
**Soluble EGF levels in incubation media from Capan-1 cells compared to media from human gallbladder myofibroblasts. **Cells were cultured in media with or without 10% FBS. Shown are the results of one experiment, which was repeated with similar results (A). B. Confocal immunofluorescence image of confluent Capan-1 cells stained for EGF.

*In vivo*, the source of EGF that drives a cancer cell to become invasive may be adjacent cancer cells, adjacent mesenchymal cells, or the cancer cell itself. In order to determine the source of EGF involved in these EGF-mediated effects on adhesion and invasion in Capan-1 cells, we looked for evidence of endogenous EGF synthesis and secretion. An ELISA for human EGF showed no soluble EGF secreted by Capan-1 cells, whereas cultured human myofibroblasts secreted soluble EGF, consistent with previous reports (Figure 10A) [22]. On immunofluorescence microscopy with an EGF antibody, however, EGF was expressed on the plasma membrane of Capan-1 cells (Figure 10B). These results suggest that Capan-1 cells synthesize but do not secrete EGF.

## Discussion

Drugs that target the EGF receptor offer promise for more effective treatment of pancreatic cancer [1]. For example, Gefitinib, a selective EGF receptor tyrosine kinase inhibitor, inhibits pancreatic cancer cell growth and invasion [23]. Understanding the mechanisms of EGF-induced cellular invasion in pancreatic cancer cells therefore is critical, as such insights could provide a means to define subpopulations of tumors more likely to be responsive to these agents and allow for more focused targeting strategies in future drug design [24]. In the current work, we have provided data to support a model in which EGF-mediated effects on de-adhesion and invasion occur via the EGF-R and erbB-2 receptor involving the PI3 kinase signalling pathway in Capan-1 cells. The involvement of the PI3-kinase pathway has also been reported in glial cell line-derived neurotrophic factor-induced migration and invasion in pancreatic cancer cells, suggesting that this signalling mechanism may be common to the invasion phenotype in pancreatic cancer cells [25]. Furthermore, we provide evidence that EGF-mediated de-adhesion and invasion are temporally linked phenomena, occurring in cells that are capable of synthesizing both EGF and EGF-R. The lack of EGF secretion by these cells suggests that the drive to invasiveness in these cells may be provided by cell-cell interactions that promote EGF-R activation, although alternative mechanisms involving EGF have not been ruled out by our results.

It is interesting to compare and contrast the results obtained by Stefani et al with our data, because these investigators used the same pancreatic cancer cell lines (Capan-1 and MIA PaCa-2), and assessed the response to EGF on adhesion on the same ECM components as in our study [26]. In our study, EGF-mediated de-adhesion in Capan-1 cells occurred with collagen I and Matrigel, and not with other extracellular matrix components. This finding is similar to results reported by Stefani et al in Capan-1 cells, although in that study, the de-adhesion response to EGF in Capan-1 cells did not reach statistical significance and no further studies involving the mechanism involved in this response were conducted. One important difference between the methodologies used in these studies was in the time course of the adhesion assay. Stefani et al treated their cells with daily EGF treatments for 5–6 days prior to performance of the adhesion assay. In contrast, our studies were performed on a much shorter time scale. Indeed, our results can be reconciled by examination of the time course results presented in Figure 5, which shows that continuous exposure to EGF for more than 32 hours leads to an abrogation of the de-adhesive response. Therefore, the de-adhesive response to EGF in Capan-1 cells reported at 6 days by Stefani et al may have been less significant than our results because the effect of EGF was more pronounced at earlier time points. A similar explanation would account for the differences in adhesion to collagen I measured with and without EGF treatment in MIA PaCa-2 cells.

The α_2_/β_1 _integrin heterodimer is the collagen I receptor, which is found in numerous tissues [27,28]. The adhesion assay results with collagen I implicated the α_2_/β_1 _integrin heterodimer, which are expressed in Capan-1 cells [29]. Higher expression of α_2 _integrin in pancreatic cancer tissue compared with normal pancreas has been reported previously [30]. However, the lack of change in expression of α_2_/β_1 _integrin with EGF treatment in Capan-1 cells suggested that functional activation of these integrins were more important than absolute expression levels in mediating the effects of EGF on adhesion. This effect of EGF differs from that described in human cutaneous squamous carcinoma cells, in which treatment with this growth factor induces expression of α_2_/β_1 _integrins and enhances adhesion and migration on type I collagen [31]. Therefore, the lack of increase in expression of α_2_/β_1 _integrin, and the de-adhesion response, are features that distinguish the EGF-induced adhesion effects in Capan-1 cells from that in squamous carcinoma cells.

The studies with phalloidin, showing changes in localization of actin microfilaments upon EGF exposure, implicate a functional effect on focal adhesion complexes as well. EGF receptor activation has been linked to focal adhesion complex changes via activation of the focal adhesion kinase, FAK [21]. As motility signalling derived from EGF-R activation required FAK [21], the de-adhesion and invasion responses noted in Capan-1 cells likely involve FAK activation also. Further experiments are necessary to prove that this link between EGF-R activation and FAK activation leading to focal adhesion complex regulation and cell motility is in fact present in Capan-1 cells as it is in other types of cancer cells [32-34].

The de-adhesive response was temporally and dose-dependently linked to stimulation of invasion in Capan-1 cells, suggesting that these responses are linked. The effects of EGF on invasion through collagen I and Matrigel were also significant, and differed from HRG-α, which did not display this effect. β_1 _integrins have been implicated to play a key role in pancreatic cancer cell invasion [35]. Furthermore, α_2 _integrins have also been implicated in EGF-induced invasion through collagen in keratinocytes [36]. Taken within this context, the finding that EGF (but not HRG-α) induces invasion through collagen I and Matrigel implicates the α_2_/β_1 _integrin in this response, acting via EGF-R and erbB2 receptors. MIA PaCa-2 cells share these properties with respect to invasion through Matrigel. Metastatic dissemination mediated by increased expression of α_2_/β_1 _integrin has been shown in ovarian carcinoma cells, so integrin-mediated invasion capabilities in Capan-1 and MIA PaCa-2 cells may also be operative [37]. In this respect, Capan-1 and MIA PaCa-2 cells also differ from breast carcinoma cells, in which re-expression of α_2_/β_1 _integrins correlates with abrogation of the malignant phenotype [38].

Recent reports have implicated the MUC4 mucin as an intramembranous ligand for erbB2 [39]. Specifically, the rat Muc4/Sialomucin complex interacts with erbB2 and induces its autophosphorylation and translocation from the basolateral to the apical membrane in CaCo-2 cells [40]. Antisense plasmid-mediated down-regulation of MUC4 expression in a pancreatic cancer cell line enhanced adhesion and decreased motility, findings that are consistent with our data [41]. While the pancreatic cancer cell line used in that study was not Capan-1, and while adhesion to and migration through plastic was assessed, the fact that the general findings between the report of Singh et al and our data are in agreement suggests that erbB-2 signalling effects on adhesion and invasion may not be limited to one particular pancreatic cell line. In this context, the contrasting results on the collagen I adhesion assay between Capan-1 cells and MIA PaCa-2 cells indicate that the de-adhesive response to EGF appears to be a feature characteristic of a metastatic cell, whereas stimulation of adhesion to collagen I by HRG-α appears to be a feature of a primary pancreatic adenocarcinoma cell. Further experiments comparing these and other pancreatic cancer cell lines would need to be performed to determine whether these differential responses to EGF and HRG-α hold true.

Finally, the finding that Capan-1 cells expressed but did not release EGF into the media has implications for the self-sustaining stimulation of the EGF-R with respect to invasion capabilities. Pancreatic cancer cells over express EGF, along with other members of the EGF family such as Cripto and epiregulin [42,43]. EGF-R, erbB-2 and related members of the EGF receptor tyrosine kinases are also over expressed in pancreatic cancer [44]. The co-expression of these EGF families of ligands and receptor tyrosine kinases suggests that an autocrine mechanism is operative in pancreatic cancer, thereby allowing autonomous activation of signalling pathways that manifests as an aggressive tumor phenotype. [4,6]. Our data suggests that, at least in Capan-1 cells, this autocrine mechanism may need to be refined so that cell-cell interactions between membrane-expressed but non-secreted EGF stimulates EGF-R on adjacent cells. Pancreatic cancer cells destined to invade extracellular matrix may therefore need to perform this function in groups rather than as individual cells.

## Conclusion

In summary, our studies on Capan-1 cells have shown that EGF-R and erbB2 receptor are likely involved in mediating a temporally linked de-adhesive and invasion response. These cellular functions involve the participation of α_2_/β_1 _integrins, which induce changes in the actin cytoskeleton at points of contact with the extracellular matrix. Finally, the autocrine nature of this growth factor signalling likely involves cell-cell interactions as EGF is synthesized but not secreted by these cells. These insights refine our understanding of the complex cellular phenomena that occur *in vivo *in the setting of pancreatic cancer invasion and metastasis, and open up avenues for further investigation into the mechanisms involved.

## Competing interests

The author(s) declare that they have no competing interests.

## Authors' contributions

AJS participated in the conception and design of the study, analyzed the data, and performed the experiments. RK participated in the conception and design of the study, analyzed the data, and wrote the manuscript. Both authors read and approved the final version of the manuscript.

## Pre-publication history

The pre-publication history for this paper can be accessed here:
